# 99th-Percentile Upper Reference Limit for the New Snibe High-Sensitivity Troponin I Assay in a Southeast Asian Population

**DOI:** 10.3390/diagnostics15192452

**Published:** 2025-09-25

**Authors:** Yun Zhang, Chin Shern Lau, Ke Yang, Soon Kieng Phua, Ya Li Liang, Tar Choon Aw

**Affiliations:** 1 Shenzhen New Industries Biomedical Engineering Co., Ltd. (Snibe), Shenzhen 518122, China; 2 Department of Laboratory Medicine, Changi General Hospital, Singapore 529889, Singapore; 3 Department of Medicine, Yong Loo Lin School of Medicine, Singapore 119077, Singapore; 4 Academic Pathology Program, Duke-NUS Graduate School of Medicine, Singapore 169857, Singapore

**Keywords:** troponins, immunoassay, acute coronary syndrome

## Abstract

**Background**: The 99th-percentile upper reference limit (99% URL) is essential for high-sensitivity cardiac troponin I (hs-cTnI) assays to diagnose acute coronary syndromes. We derived the gender-specific 99% URLs for the new Snibe hs-cTnI assay and verified its high-sensitivity performance. **Methods**: The Snibe hs-cTnI assay has a claimed limit of blank/detection/quantitation of 0.5/1.0/2.0 ng/L, a precision of 5.67% (@ 9.83 ng/L), 4.65% (@ 21.0 ng/L) and 3.77% (@ 174 ng/L), an analytical measuring range of 1.00–500,000 ng/L, and a claimed 99% URL of 20.1/11.8/17.5 ng/L (males/females/overall). Assay precision was evaluated using kit control materials. A total of 846 (M 444, F 402) anonymized leftover samples from healthy individuals were assessed for the derivation of 99% URLs. **Results**: The inter-assay CV was 6.2/4.4% (@ 10.1/20.6 ng/L). The 10/20% CV corresponded to a concentration of 5.2/3.0 ng/L. The assay recorded detectable hs-cTnI values in 64% of women and 70% of men. Hs-cTnI values were significantly lower in females than males (females vs. males median 2.6 vs. 3.2 ng/L, Mann–Whitney *p* = 0.005). The derived 99% URL for the population was 7.7 ng/L (90% CI 6.93–22.8 ng/L) for women and 13.7 ng/L (90% CI 10.6–41.4 ng/L) for men. When confined to subjects ≥40 years old only, males had a 99% URL of 14.8 ng/L (*n* = 412, 90% CI 10.6–41.4 ng/L) and 8.1 ng/L (*n* = 377, 90% CI 6.95–22.8 ng/L) for females, respectively. **Conclusions**: The Snibe hs-cTnI assay fulfills the requirements of a hs-cTn assay. Gender-specific 99% URLs are derived for this assay and they increased with age.

## 1. Introduction

The 99th-percentile upper reference limit (99% URL) of troponin is recommended as a cut-off to diagnose acute coronary syndromes by the fourth universal definition of myocardial infarction [1]. The 99% URL remains the recommended diagnostic threshold for myocardial injury and myocardial infarction by both the International Federation of Clinical Chemistry [2] and cardiology specialty societies [3]. In addition, it is increasingly recognized that the troponin 99% URL may serve other functions, for example, in the prognostication of patients with myocardial infarction, where nearly 50% of patients with 0 h troponins above the 99% URLs may experience poorer outcomes (rehospitalization, death, non-fatal myocardial infarction or revascularization) compared to 70% in patients who did not exceed that threshold [4]. Even in asymptomatic individuals, hs-cTnI has been shown to effectively predict cardiovascular disease, for example, in the BiomarCARE project, where a threshold of 6 ng/L had a hazard ratio of 1.63 for all cause mortality, and a hazard ratio of 2.6 for cardiovascular death in community living subjects between the age of 20–99 years (mean age 52.2 years) [5].

New high-sensitivity cardiac troponin (hs-cTn) I and T assays have a better negative predictive than older assays, and facilitate the rapid rule—out of myocardial injury/infarction [6]. Algorithms based on these newer troponin assays have been shown to effectively decrease the length of stay in some emergency departments by up to 2.2 h [7], with a single hs-cTn measurement ruling out 24.1% of chest pain patients, and increasing to 63.8% after the second measurement 1 h later. Indeed, less than 15% of patients required hospital transfer after clinic assessment, which greatly reduced the burden of care in the emergency department. These algorithms have also proven levels of safety. In patients discharged from the emergency department using a 0/1 h algorithm, only 0.45% experienced morbidity/mortality within 30 days, compared to 0.61% in control hospitals [8]. Indeed, when the diagnostic performance of a new hs-cTnI assay (Hybiome hs-cTnI assay, correlation with Beckman hs-cTnI r = 0.926) was assessed using four different methods (limit of detection cut-off, 99% URL cut-off, 0/1 h algorithm, 0/2 h algorithm for the diagnosis of NSTEMI), the single 99% URL cut-off had a negative predictive value/accuracy of 98.48/85.4%, whereas the 0/2 h algorithm provided slightly better performance with negative predictive value/accuracy of 98.5/88.6% [9].

However, there remains substantial variability in the thresholds used for the 99% URL. In fact, in a study of 276 participating institutions, only a third followed the guidelines to use the 99% URL decision levels in the diagnosis of myocardial infarction [10], among 21 unique troponin assays from 9 manufacturers. The picture becomes even further muddied with the introduction of newer point-of-care troponin testing, especially with the recent introduction of point-of-care troponin assays that can fulfill the criteria for high-sensitivity. For example, the Siemens Atellica hs-cTnI point-of-care assay demonstrated excellent sensitivity (98.9%) and negative predictive values (99.5%) when compared to an Abbott hs-cTnI assay [11], and also when compared to a Beckman hs-cTnI assay: Siemens/Beckman ROC areas were 0.94/0.97. These point-of-care tests will also require the use of specific 99% URL diagnostic thresholds. Already efforts are being made to try to determine cut-offs for their use in rapid rule-out algorithms [12].

The 99% URL should be derived from a reference cohort of healthy individuals, using a minimum of 800 subjects (400 subjects per gender) to ensure statistical power [2]. There are some advantages in the use of hs-cTnI, as it is less affected by skeletal muscle disorders. In one study of patients with muscular disease (including myotonic dystrophy, dermatomyositis, myasthenic syndromes) [13], hs-cTnT assay concentrations were much higher in patients with skeletal muscle disorders (median 16 ng/L vs. 5 ng/L in controls), compared to hs-cTnI (2.5 ng/L vs. 2.9 ng/L in controls), with up to 55% of muscular disease patients having a higher hs-cTnT than the upper limit of normal. In another study of 18 patients with myopathy, 17 had increased cTnT values with no increase in cTnI [14]. A similar trend was found in subjects with statin induced myopathy [13,15], with median cTnT increasing to 0.19 ug/L on presentation despite no increase in cTnI. This can cause confusion when using hs-cTnT in the clinical setting, as up to 15.3% of patients with myopathy would be mistakenly ruled-in for myocardial infarction based on European Society of Cardiology algorithms [16]. However, both troponins T and I are increased in chronic kidney disease (CKD), but less so with troponin I [17]. In a study of 119 patients with a wide range of estimated glomerular filtration rates (eGFR), hs-cTnT demonstrated a strong relationship with decreasing eGFR (R^2^ = 0.625), whereas no significant relationship with hs-cTnI was found (R^2^ = 0.013) [18]. The effect of CKD on the accuracy of a single hs-cTnI at presentation can be quite stark: hs-cTnI <5 ng/L at presentation identified only 17% of patients with CKD as low risk for myocardial events, as opposed to 56% without renal impairment, with positive predictive values and specificity at the 99% URL lower in renal impairment (50% and 70.9% vs. 62.4% and 92.1% in non-CKD subjects) [19]. Indeed, the use of serial changes in cardiac troponin concentration is essential in the assessment of myocardial disease in patients with chronic kidney disease [20].

Snibe has recently introduced its latest generation of hs-cTnI assay for its automated chemiluminescence immunoassay system, with reportedly greater selectivity and less interference. As the 99% URL is essential for the use of any troponin result, we sought to derive gender-specific 99% URLs for this new hs-cTnI assay.

## 2. Materials and Methods

### 2.1. The Snibe hs-cTnI Assay

The Snibe hs-cTnI assay (Shenzhen New Industries Biomedical Engineering Co., Ltd. (Snibe), Shenzhen, Guangdong, China) is a sandwich immunoassay, with a claimed limit of blank/detection/quantitation of 0.5/1.0/2.0 ng/L, a claimed precision of 5.67% (9.83 ng/L), 4.65% (21.0 ng/L) and 3.77% (174 ng/L), and a linear range of 2.00–50,000 ng/L, an analytical measuring range of 1.00–500,000 ng/L (see Table 1). It has a throughput of 180 tests/hour, and an assay time of 21 min. The sample, magnetic microbeads coated with cTnI monoclonal antibody, N-(-4-aminobutyl)-N-ethylisoluminol (ABEI) labeled with another cTnI monoclonal antibody and buffer are mixed thoroughly. Following incubation, the reactants form sandwich complexes. In designing the assay, coating/labeling cTnI antibody pairs were selected with the highest signal-to-noise ratio. A three-step strategy of signal-to-noise ratio screening, interference assessment (TNNI1, TNNI2 and heparin), and clinical sample validation was adopted for 12 antibodies covering four epitopes of cTnI (24–40, 41–49, 87–91, 171–191 aar). The optimal antibody combination was finally determined: the capture antibody targeting 41–49 aar, and the detection antibody targeting 24–40 aar and 87–91 aar. The optimal reaction system parameters were determined through single-factor optimization: magnetic bead dilution ratio of 1.0 mg/mL, ABEI-labeled antibody dilution ratio of 1:500, 50 μL sample loading volume, and 10 min of incubation time. After precipitation in a magnetic field, the supernatant is decanted followed by a wash cycle. Subsequently, a starter solution is added to initiate a chemiluminescent reaction. A composite blocking system was also employed during the coating process of the magnetic particles, integrating macromolecular and small-molecule blockers to reduce non-specific binding. A 100 μg/mL heterophilic antibody blocker is also added to inhibit human anti-mouse antibody and rheumatoid factor interference (interference rate <5%). In addition, this detection system adopted an improved ABEI label, uniform-sized magnetic microspheres, and a composite blocking system, combined with a dual-labeled antibody strategy. This has enhanced the reagent’s anti-interference ability while ensuring detection sensitivity, providing a reliable solution for rapid and accurate clinical hs-cTnI detection. It has a claimed 99% URL of 11.8 ng/L for females, and 20.1 ng/L for males, derived from 617 apparently healthy individuals in China.

### 2.2. Assay Precision Analysis and Derivation of the 99% URL

Assay precision was evaluated according to CLSI EP5-A2 protocols [21] using the assay’s internal quality control material.

### 2.3. Derivation of the 99% URL

We analyzed anonymized leftover samples from a population of 866 healthy individuals (no history of diabetes, renal impairment, or cardiovascular disease), with 846 subjects (M = 444, F = 402) in the final population after excluding outliers (Tukey method). All subjects had normal renal function (eGFR > 90 mL/min) and no history of heart disease. Visibly hemolyzed or turbid samples were excluded. We subsequently derived the 99% URL (99% right sided interval, non-parametric, following CLSI guidelines [22] for percentiles and their CIs) for males and female hs-cTnI.

### 2.4. Statistical Analysis

We used MedCalc Statistical Software (version 20.008, MedCalc Software Ltd., Ostend, Belgium) for statistical analyses.

As this investigation was part of routine clinical laboratory service evaluation/population surveillance, national/hospital regulations exempt such testing from institutional board review.

## 3. Results

### 3.1. Assay Performance

**Figure 1:**
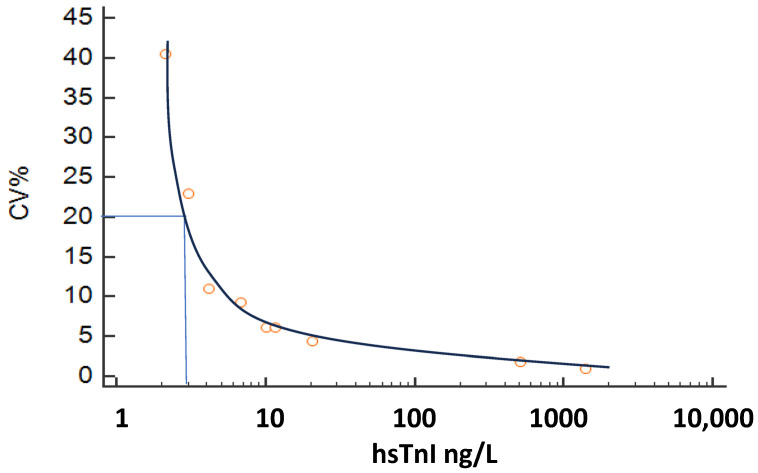
Imprecision profile of the Snibe hs-cTnI. 20% CV at 3.0 ng/L.

The inter-assay CV of internal quality control materials was 6.2% (@ 10.1 ng/L) and 4.4% (@ 20.6 ng/L). From the inter-assay precision profile, the 10/20% CV corresponded to a concentration of 5.2/3.0 ng/L (see Figure 1). The assay recorded detectable hs-cTnI values more than the limit of detection in 64% of women and 70% of men. In a repeat analysis at another center, CV% was noted to be 32.4/11.7/6.6/5.6/2.4/2.7% (@1.1/2.1/3.3/4.6/5/5/6.6 ng/L), with a 10% CV at 2.46 ng/L and a 20% CV at 1.53 ng/L. It is recommended that hs-cTn assays should not report values below the 20% CV limit of quantitation, and as such, results <3.0 ng/L on this assay would be reported as <3.0 ng/L.

### 3.2. Population hs-cTnI 99% URL

**Figure 2:**
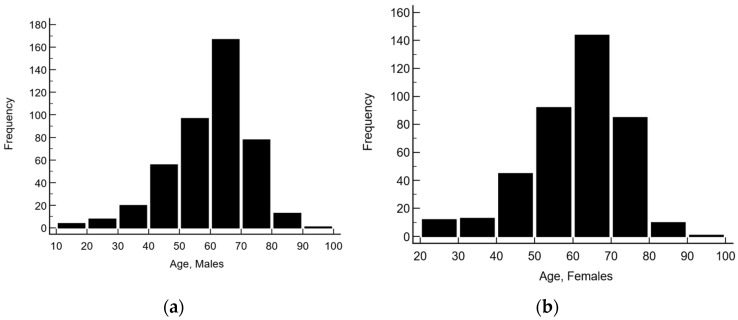
Age distribution of (**a**) males and (**b**) females.

The age of subjects ranged from 18 to 94 years (mean 60.1 ± 12.7 years). Males were from 18 to 94 years (mean 59.7 ± 12.8 years), and females were 20–90 years (mean 60.5 ± 12.6 years) (see Figure 2).

**Figure 3:**
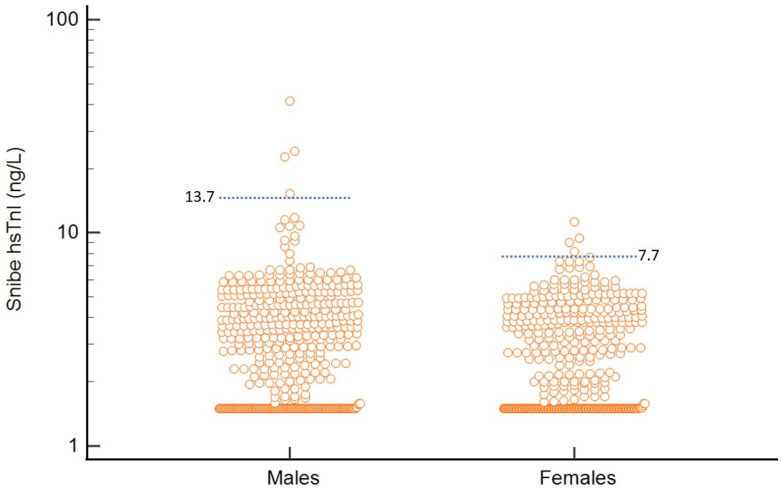
Distribution and 99% URL of Snibe hs-cTnI in females vs. males in the total population.

Median hs-cTnI of the study population was 2.9 ng/L (SD 2.6 ng/L) (Kolmogorov–Smirnov test D = 0.24, normality of distribution rejected). For females, the hs-TnI values were significantly lower than males (median: females vs. males 2.6 vs. 3.2 ng/L, Mann–Whitney *p* = 0.005) (see Figure 3). The derived 99% URL for the population was 7.7 ng/L (90% CI 6.93–22.8 ng/L) for women and 13.7 ng/L (90% CI 10.6–41.4 ng/L) for men. When we restricted analysis to only subjects ≥40 years old, males had a 99% URL of 14.8 ng/L (*n* = 412, 90% CI 10.6–41.4 ng/L) and females had a 99% URL of 8.1 ng/L (*n* = 377, 90% CI 6.95–22.8 ng/L).

## 4. Discussion

The Snibe hs-cTnI assay demonstrated excellent performance and fulfilled the necessary criteria to confirm its status as a high-sensitivity assay (detectable hs-cTnI values > LOD in 64% of women and 70% of men, with a CV of 10% at 5.2 ng/L). Although our 99% URLs differ from the manufacturer’s values (males 13.7 vs. 20.1 ng/L, females 7.7 vs. 11.8 ng/L), these may be attributed to demographic/geographical differences between the reference populations: the manufacturer information stated that their 99% URLs were derived from 617 apparently healthy individuals in China; however, we have no information on the eGFR, age and distribution of said subjects (all our subjects had eGFR >90 mL/min). In addition, we do not have information on assay lot differences, calibration methods or statistical methods used to derive the manufacturer’s proposed 99% URLs, and it is possible that these may have further contributed to the above differences (our study involved one lot of reagents). This highlights the importance of verifying the applicability of manufacturer 99% URLs prior to usage, and it is highly recommended that population specific cut-offs are derived from healthy local populations prior to hs-cTn assay use and moreover, inclusion of a younger cohort of healthy individuals or subjects with eGFRs of only >90 mL/min would further decrease the 99% URL. In addition, our derived 99% URLs using this assay were higher in males and older subjects, which is similar to findings from other studies reporting 99% URLs for hs-cTnI assays [23].

Defining the 99% URL from a healthy cohort is a contentious issue, as the definition of a healthy cohort is not standardized, and can be affected by the age and renal function of the selected population. Naturally, reference cohorts need to exclude major comorbidities such as known cardiovascular disease, treatment for hyperlipidemia/hypertension, diabetes, chronic renal disease, abnormal BMI, smoking and any other chronic condition that could affect the myocardium. However, by incorporating subjects of all ages (usually 18 years old and above), results can be skewed towards the younger population that tends to be healthier with less comorbidities than older individuals. Furthermore, it has been shown that troponin levels increase in a step-wise fashion with age [24,25]. From small countries like Singapore, to larger nations like the UK, cardiovascular disease occurs overwhelmingly in older patients > 40 years old [26,27]. In Singapore, the overall median age of onset of ACS was 71.4 years old in 2022, and only <2% of reported cases were <40 years old. This effect would make a 99% URL derived from a skewed younger cohort much more likely to cause a larger number of false positives. Indeed, some studies [28] do show that the influence of age can be more pronounced in males, increasing by a greater degree in men aged 40–44 years old (male/female median cTnI 2.7/2.1) vs. 60–65 years old (male/female median cTnI 3.4/2.7). This is also seen in our study, where the 99% URLs increased in males to a greater degree when we restricted our analysis to subjects ≥ 40 years old (males 13.7 to 14.8 ng/L, females 7.7 to 8.1 ng/L). As such, a reasonable age distribution should still be sought in studies that attempt to derive the 99% URL for hs-cTns, with special effort to include sufficient numbers of older subjects. Indeed, age also seems to affect the prognostic values of cTnI, with predictive ability of cTnI dropping after 50 years old from an odds ratio for myocardial infarction decreasing from 9.4 when <30 years old to only 3–5 after 50 years old [29]. One strength of our study was the inclusion of more older subjects to generate the 99% URL; the mean age of our overall population was 60 years old, with a good balance of males (53%) and females. Another strength is that we have used a sufficiently large population (>800 subjects) with >400 subjects in each gender for sufficient statistical power [2].

Undoubtedly, gender-specific hs-cTn 99% URLs should be determined and reported. The fact that females have generally lower hs-cTn values is well established for both hs-cTnI and hs-cTnT [30,31,32]. Indeed, in a recent systemic review [33], 19 studies deriving gender-specific 99% URLs for 11 different hs-cTnI assays found lower female 99% URLs compared to male and overall 99% URLs. This is expected given that women have smaller cardiac mass than men [17]. In addition, studies have also shown that hs-cTnI can be even more strongly associated with ACS in females, with increased hazard ratios at a hs-cTnI of 10ng/L in females than males (9.7 vs. 5.6 in females vs. males) in 19,501 subjects [30]. In the light of these caveats to the 99% URL, some studies have suggested the use of alternative strategies to rule-in ACS, including combined tools incorporating age, sex and paired cTn measurements to generate risk scores, or other novel algorithms [34]. However, these modifications have yet to be more widely validated and adopted. Further efforts would be required to encourage the establishment and usage of gender-specific 99% URLs to rule-out myocardial infarction.

In addition to age and gender, the other most common factor that affects cTn is renal function [20]. This is an important issue, as even minor levels of renal impairment can significantly affect cTn levels, and subsequently the derivation of the 99% URL: in one Danish study [35], 99% URLs were lower with an eGFR cut-off of ≥90 mL/min/1.73 m^2^ than if a cut-off of 60 mL/min/1.73 m^2^ was used in the reference population, and this was consistent across three analyzers (Siemens/Abbott/Roche 99% URL was 46/14/10 ng/L if eGFR ≥ 90, 70/18/13 if eGFR 60). This is supported by other studies [25,31] that show a consistent step-wise increase in cTns with decreasing renal function even with a drop from eGFR 90 to 60. This in turn will interfere the diagnostic performance of 0/2 h algorithms for ruling in myocardial injury. Compared to patients with CKD, positive predictive values at hs-cTnT thresholds at >200/300 ng/L was 73/80%, compared to 59/54% in patients with CKD [36]. Thus, we only included subjects with an eGFR ≥ 90 mL/min/1.73 m^2^ in our reference population in order to account for these effects.

One limitation of our study is that our reference population was from a single geographical area (Southeast Asia) and as such, results may not be generalizable to other populations. In addition, we have yet to compare the performance of the Snibe assay to other platforms, and further studies would be required. However, it must still be noted that hs-cTnI assays are not standardized or harmonized [37], in part because of the extremely varied capture/detection antibodies between manufacturers. For example, detection methods using antibodies targeting the cTnI carboxyl-terminal region can exhibit altered reactivity with cTnI fragments [38], leading to falsely elevated values. In a recent study of 9 hs-cTnI assays [32] there was extensive variation between assays even with gender-specific 99% URLs, with hs-cTnI 99% URLs ranging from 8 to 68 ng/L in men and 3–40 ng/L in women. When diagnostic power is compared between the 99% URLs of different assays [4], variation was also noted in performance, with ROC curve areas varying from 0.874 to 0.897, with varying sensitivities ranging from 87.3 to 98.6% (when using a unisex 99% URL). This variation becomes even more problematic in patients with severe CKD, in part due to decreased renal clearance of troponins, chronic inflammation in CKD, and uremic toxins like homocysteine that contribute to atherosclerosis and myocardial damage [39]. As such, when in doubt, serial measurements of hs-cTn are strongly recommended, especially in patients with CKD, as the deltas based on the same assay would be the most helpful in ruling out myocardial injury [40].

While the investigation of biological interference for the Snibe hs-cTnI assay was beyond the scope of the current study, we did exclude outliers with high hs-cTnI values. Hs-cTnI assays are also more prone to the effects of macrotroponin, resulting in falsely elevated troponin results in the absence of myocardial injury [41]. Notably, the prevalence of macrotroponin I can be quite high, with reported values of between 5 and 52% in community studies, especially in patients with hs-cTnI concentrations above the 99% URL, with macrotroponin I affecting results across platforms [42,43]. Indeed, diagnostic performance was comparatively better in patients without macrotroponin than those with macrotroponin (in the Beckman hs-cTnI assay, ROC in patients with macrotroponin vs. without was 0.576 vs. 0.8338, *p* = 0.0006) [43]. Furthermore, the effects of macrotroponin on the 99% URLs can potentially be serious. In one study of healthy individuals who had previously been used to generate 99% URLs, macrotroponin I was found to be present in 76% of high hs-cTnI samples, and after exclusion of these samples, 99th URLs decreased from 117 ng/L to 22 ng/L in men, and 37 ng/L to 9 ng/L in women for the Siemens Atellica assay. Indeed, the hs-cTnT comparator in this study had no cases of macrotroponin [44]. Thus, macrotroponin can potentially severely affect the classification rates of myocardial events. However, a limitation of our study is that we did not investigate for macrotroponin in our population. The prevalence of macrotroponin in the Snibe assay in our cohort is unknown, which may potentially affect final results. Although we excluded outliers (Tukey), further investigation on the effects of macrotroponin (complexes formed between auto-antibodies and troponin I) will need to be undertaken in the future for the Snibe assay in our population.

## 5. Conclusions

In conclusion, we have successfully derived gender-specific 99% URLs for the new Snibe hs-cTnI assay, and established the high-sensitivity status of the assay. Further studies would be required to determine rule-out/in cut-offs for this new assay, as well as the clinical performance of the assay in the diagnosis of myocardial infarction.

## Figures and Tables

**Table 1:** Comparison of previous and new generation of the Snibe hs-cTnI assay.

	Previous Generation	Current Assay
99th Percentile (ng/L)	<10	Female 11.8, male 20.1, overall 17.5
Precision (%)	7.13/6.62 (99.024/310.045)	5.67/4.65/3.77 (9.831/21.028/174.294)
Limits of Blank/Detection/Quantitation (ng/L)	1.0/1.5/3.0	0.5/1.0/2.0
Measuring Range (ng/L)	1.0–50,000	1.00–500,000

## Data Availability

The datasets generated during and/or analyzed during the current study are not publicly available due to privacy issues and national laws but are available from the corresponding author on reasonable request under the provision that data may not leave the hospital/center premises.

## Abbreviations

The following abbreviations are used in this manuscript:

99% URL	99th-percentile upper reference limit
Hs-cTnI/T	High-sensitivity cardiac troponin I/T
eGFR	Estimated glomerular filtration rate
CKD	Chronic kidney disease
ABEI	N-(-4-Aminobutyl)-N-ethylisoluminol
